# Matrix Metalloproteinases in Cerebral Vasospasm following Aneurysmal Subarachnoid Hemorrhage

**DOI:** 10.1155/2013/943761

**Published:** 2013-04-03

**Authors:** Vivek Mehta, Jonathan Russin, Alexandra Spirtos, Shuhan He, Peter Adamczyk, Arun P. Amar, William J. Mack

**Affiliations:** Department of Neurosurgery, University of Southern California, 1200 North State Street, Suite 3300, Los Angeles, CA 90033, USA

## Abstract

Delayed cerebral vasospasm is a significant cause of morbidity and mortality following aneurysmal subarachnoid hemorrhage (SAH). While the cellular mechanisms underlying vasospasm remain unclear, it is believed that inflammation may play a critical role in vasospasm. Matrix metalloproteinasees (MMPs) are a family of extracellular and membrane-bound proteases capable of degrading the blood-rain barrier (BBB). As such, MMP upregulation following SAH may result in a proinflammatory extravascular environment capable of inciting delayed cerebral vasospasm. This paper presents an overview of MMPs and describes existing data pertinent to delayed cerebral vasospasm.

## 1. Background

Delayed cerebral vasospasm is a devastating complication of subarachnoid hemorrhage (SAH). It typically occurs within fourteen days of aneurysmal rupture, and it is associated with significant morbidity and mortality [1, 2]. While pathophysiology remains incompletely understood, the interplay between inflammation and the innate immune response is strongly implicated. 

Following SAH, increased blood-brain barrier (BBB) permeability engenders a proinflammatory milieu in the cerebral cisterns and extravascular space. Subarachnoid blood initiates leukocyte transmigration via cellular margination, adhesion, rolling, and diapedesis [3]. The process necessitates violation of the tight junctions between endothelial cells of the BBB and typically occurs in response to stimulatory chemoattractants or chemokines. Coupled with concurrent physiologic derangements, these molecular alterations can incite delayed cerebral vasospasm.

Regulation of the extracellular matrix and basal lamina by matrix metalloproteinase (MMP) enzymes may play a critical role in vasospasm. MMPs have been studied extensively in the pathogenesis of ischemic stroke and the development of aortic and cerebral aneurysms [4]. Recently, investigations have examined the role of MMPs in the setting of SAH. In this paper, we specifically review the function of MMPs in cerebral vasospasm. Understanding the complex interactions between inflammation and degradation of the extracellular matrix may ultimately allow for better development of diagnostic markers and targeted therapies relevant to the management of delayed cerebral vasospasm. 

## 2. MMP Review

MMPs are a family of extracellular and membrane-bound proteases capable of degrading or proteolytically modifying the extracellular matrix (ECM) through interactions with collagenases, laminins, and proteoglycans [5]. They utilize zinc-dependent endopeptidases to regulate physiologic processes relevant to development, homeostasis, and tissue modeling [6]. MMPs are secreted by macrophages, leukocytes, smooth muscle cells, endothelial cells, astroglia, and microglia in response to growth factors and inflammatory cytokines [7–12]. 

The expression and activity of MMPs are highly regulated. Activation through the zymogen in a cysteine switch was first demonstrated by Van Wart and Birkedal-Hansen [13]. Once activated, MMPs can proteolyse other pro-MMPs, enabling rapid multiplication. Yong et al. demonstrated that MMP-3 is capable of activating pro MMP-1, MMP-7, MMP-8, MMP-9, and MMP-13, while MMP-2 can activate pro MMP-1, MMP-9, and MMP-13 [5]. By contrast, MMP-9 has not been shown to activate other MMPs. Activated MMPs have important roles in neuronal development, function, and differentiation [14–18]. 

Inhibition is mediated by a family of proteins known as tissue inhibitors of metalloproteinases (TIMPs) [19]. TIMPs utilize high affinity, noncovalent catalytic site binding to functionally inhibit MMPs [20]. Activated leukocytes are a major source of MMP production following brain injury [21, 22]. As MMPs degrade the basal lamina, cellular transmigration is facilitated. These processes may work in concert to affect a positive feedback loop. Degradation of the blood-brain barrier allows leakage of inflammatory mediators into the extracellular matrix. The proinflammatory environment, in turn, may promote arterial dysregulation, stimulate leukocyte migration, enhance cytokine/chemokine production, and render the affected vessels susceptible to narrowing. A review of the specific MMPs follows; only those that have been studied in the setting of delayed cerebral vasospasm (MMP-1, MMP-2, MMP-8, MMP-9, and MMP-13) are included. Majority of the data focuses on MMP-9.

### 2.1. MMP-1

Studies have demonstrated that MMP-1 mediates collagen lattice contraction. The effect is durably negated by MMP inhibitors [23–25]. To date, a single investigation has examined the role of MMP-1 in cerebral vasospasm. In an experimental rodent model, Satoh et al. utilized immunohistochemical and western blot analysis of basilar arteries to quantify MMP-1 deposition. When compared to controls, the SAH cohort demonstrated increased MMP-1, primarily localized to the smooth muscle cytoplasm. This elevation peaked at 30 minutes post-SAH and was temporally concordant with vessel narrowing [23]. 

### 2.2. MMP-2

Only one group has examined the role of MMP-2 in the pathogenesis of cerebral vasospasm. Horstmann et al. compared serum MMP-2 levels in 11 SAH patients and 20 normal controls [7]. They demonstrated persistently low MMP-2 levels in the SAH cohort throughout the study period. Other investigators, however, have documented elevated MMP-2 levels in vessel walls of ruptured and unruptured cerebral aneurysms [26–28] and in patients who suffered ischemic stroke [29]. Due to a paucity of large studies and conflicting data, the precise role of MMP-2 in the development of cerebral aneurysms and pathogenesis of vasospasm remains unclear. 

### 2.3. MMP-9

The putative role of MMP-9 in cerebral vasospasm was first explored by McGirt et al. [30]. The authors examined serum concentrations in thirty-eight SAH patients. They compared levels with individuals admitted for elective aneurysm clipping and patients who suffered from non-SAH stroke. Further, they assessed MMP-9 levels in SAH patients who developed cerebral vasospasm and those who did not. Onset of vasospasm was defined as transcranial Doppler flowmetry velocities greater than 180 cm/s and/or new focal neurological deficits with angiographically confirmed vasospasm. 57% of SAH patients developed cerebral vasospasm. Mean peak serum MMP-9 levels were increased in the SAH cohort that developed vasospasm when compared to the group that did not. Further, peak MMP-9 levels for the SAH cohort that developed vasospasm were significantly elevated when compared to the focal ischemia and control cohorts. MMP levels of greater than 700 ng/ml independently increased the odds of subsequent vasospasm by 25-fold [30]. These findings first advocated MMP-9 as a potential diagnostic biomarker for cerebral vasospasm in the setting of SAH and incited significant interest in related research.

In a rat SAH model, Sehba et al. examined the role of MMP-9 on basal lamina integrity in the cerebral microvasculature [31]. The authors noted colocalization of MMP-9 upregulation and collagen IV degradation [31]. Collagen IV is a major structural component of the basal lamina, comprising approximately 90% of the total protein [32, 33]. They concluded that MMP-9 may play a role in BBB permeability [31]. Vikman et al. examined the genetics of MMP upregulation in the setting of SAH. The authors noted activation of four key signal transduction molecules (p38, ERK1/2, Elk-1, and activating transcription factor-2) and resultant transcription of ECM-related (MMP8, MMP9, and MMP13) and inflammatory (IL6, TNFa, IL1b, CXCL1, CXCL2, CCL20, and iNOS) genes [34]. The findings suggested one signal transduction pathway by which MMPs are activated in response to SAH and elucidated potential targets for therapy.

Horstmann et al. examined serum MMP-9 concentrations in a relatively small cohort of SAH patients and compared values to normal controls [7]. The authors found MMP-9 levels to be increased in the SAH group, but they failed to demonstrate correlation with clinical Hunt and Hess scores. The temporal pattern of MMP-9 elevation was concordant with that of clinical vasospasm [7]. Feiler et al. examined the influence of MMP-9 on cerebral edema and functional outcomes following SAH in MMP-9 knockout (MMP-9^−^/^−^)mice [1]. They found no significant differences in acute intracranial pressure (ICP), cerebral blood flow, or mean arterial blood pressure between MMP-9^−^/^−^ and wild-type mice. However, day 7 mortality was significantly lower in MMP-9^−^/^−^ mice (*P* = 0.03; 20% versus 60%). The MMP-9 knockout mice also demonstrated less brain edema, lower chronic ICP, and improved neurological recovery when compared to the wild-type mice. Findings suggest a role for MMP-9 in the genesis of cerebral edema and increased intracranial pressure following SAH [1]. 

Early brain injury (EBI) is believed to cause significant morbidity and mortality following SAH. Guo et al. explored the potential role of MMP-9 in this process. The study examined ECM protein lamina integrity and anoikis/apoptosis in the hippocampal region [35]. The investigators determined that MMP-9 activity is directly correlated with number of apoptotic neurons. Injury increased from 12 to 72 hours, with peak activity evident at 24 hours. Similarly, laminin density decreased at 12 hours, reached a nadir at 24 hours, and began to recover at 48 hours. Brain water content, a surrogate measure for cerebral edema, increased at time points relevant to neuronal apoptosis. The authors concluded that MMP-9 plays an important role in the pathophysiology of early brain injury following SAH via degradation of laminin and resultant anoikis of hippocampal neurons [35].

A single long-term study has associated MMP-9 levels and inflammation with poor clinical outcomes following SAH. Chou et al. examined MMP-9 and neutrophil levels in blood and CSF of fifty-five SAH patients [21]. Samples were acquired between postbleed days 0 and 14. The investigators examined associations between the following parameters: (1) serum and CSF leukocyte and neutrophil and MMP-9 levels, (2) development of cerebral vasospasm, and (3) poor clinical outcome (three-month modified Rankin score) [21]. Development of cerebral vasospasm was independently associated with persistent elevation of blood leukocytes and neutrophils on post-SAH days 0–14 but not with CSF or serum MMP-9 levels. Poor three-month modified Rankin scores correlated with elevated blood neutrophil counts on post-SAH days 2-3, elevated serum MMP-9 levels on post-SAH days 4-5, and elevated CSF MMP-9 values on post-SAH days 0–14. Further, blood neutrophil levels correlated with MMP-9 levels on post-SAH days 6–8 and 10–14 [21]. Together, these findings suggest that neutrophils may represent a major source of MMP-9 production in the early post-SAH time period. As MMP-9 levels were associated with poor three-month outcomes, but not delayed cerebral vasospasm, the authors postulate a role for MMP-9 in alternate mechanisms of brain injury (aside from vasospasm) following SAH [21]. 

Wang et al. recently described the time course of basilar artery MMP-9 expression in an experimental murine model of SAH [36]. Further, the investigators explored the effect of SB-3CT, a selective MMP-9 inhibitor, on the development of cerebral vasospasm. They discovered that expression of MMP-9 peaked on postbleed day 3 and returned to normal by day 14 which is consistent with the time course of delayed cerebral vasospasm. The authors noted a significant reduction in vasospasm (measured by cross-sectional area of the basilar artery) on postbleed day 3 following the administration of SB-3CT [36]. These findings highlight an MMP-9 inhibitor which may hold therapeutic promise in the setting of cerebral vasospasm. 

## 3. MMP Inhibitors

Recent studies have assessed the utility of MMP inhibitors in translational models of stroke and cerebral ischemia. However, few inhibitory strategies have been investigated in the setting of vasospasm. In a study by Dittmar et al., various classes of MMP inhibitors including monoclonal antibodies, tissue MMP inhibitors, and broad-spectrum MMP inhibitors (BB-94, BB-1101, and KB-R7785) proved to be effective in reducing tissue damage in an animal model of cerebral ischemia [37]. In a rodent model, Reijerkerk et al. demonstrated that BB-3103, a broad-spectrum matrix metalloproteinase (MMP) inhibitor, decreased endothelial gap formation, occludin loss, and the ability of monocytes to traverse the endothelium [38]. Future studies may seek to translate these positive findings from closely related systems into relevant cerebral vasospasm models.

## 4. Conclusion

MMPs are a family of proteases that preserve the structure of blood vessels. Under normal conditions, they regulate transfer of molecules, aid in adaptation of the ECM, and maintain the BBB. In state of disease, they can mediate breakdown of the BBB and thus modify the local inflammatory response. Together, these factors may incite or propagate delayed cerebral vasospasm. In this capacity, MMPs likely contribute to progressive injury following ischemia and aneurysmal rupture. MMPs could ultimately provide a potential biomarker for early diagnosis or a target for therapeutic intervention in the setting of delayed cerebral vasospasm following SAH.
